# Evidence for the Adaptive Learning Function of Work and Work-Themed Play among Aka Forager and Ngandu Farmer Children from the Congo Basin

**DOI:** 10.1007/s12110-018-9314-6

**Published:** 2018-04-30

**Authors:** Sheina Lew-Levy, Adam H. Boyette

**Affiliations:** 1 Department of Psychology, University of Cambridge, Cambridge, CB2 3RQ UK; 2 Duke University, Box 90025, Durham, NC 27708 USA

**Keywords:** Hunter-gatherers, Evolution of childhood, Social learning, Play, Gender bias, Ethnic bias, Embodied capital theory

## Abstract

Work-themed play may allow children to learn complex skills, and ethno-typical and gender-typical behaviors. Thus, play may have made important contributions to the evolution of childhood through the development of embodied capital. Using data from Aka foragers and Ngandu farmer children from the Central African Republic, we ask whether children perform ethno- and gender-typical play and work activities, and whether play prepares children for complex work. Focal follows of 50 Aka and 48 Ngandu children were conducted with the aim of recording children’s participation in 12 categories of work and work-themed play. Using these data, we test a set of hypotheses regarding how age, gender, ethnicity, and task complexity influence children’s activities. As hypothesized, we find performance of work-themed play is negatively correlated with age. Contrary to our hypothesis, children do not play more than they work at complex tasks, but they work more than they play at simple ones. Gender and ethnicity are associated with play and work at culturally salient activities, despite availability of other-gender and other-ethnicity social partners. Our findings show that ethnic and gender biases are apparent in the play and work behavior of Aka and Ngandu children. Moreover, our results show that play helps both forager and farmer children learn complex skills, consistent with play having an adaptive learning function.

Since childhood is an important time for acquiring cultural knowledge, studying how children learn has important implications for understanding the evolution of extended childhood in humans (Crittenden 2016; Flynn et al. 2013; Kaplan and Robson 2002; Konner 2005, 2010). Many mammals, including humans, spend a large proportion of their juvenile years in play; thus, studying how play contributes to developing adult competencies is a fruitful avenue through which to study learning (Pellegrini and Bjorklund 2004; Smith 1982). However, researchers still debate whether play has an adaptive function (Bock 2002, 2005; Bock and Johnson 2004; Byers and Walker 1995; Crittenden 2016). If play functions as an adaptive form of learning, then play should prepare children for survival and reproduction. However, in many small-scale societies, children learn via productive work throughout childhood (Chick 2009; Ember and Cunnar 2015; Lancy 2012, 2016; Munroe et al. 1984; Whiting and Whiting 1975). This begs the question: if children can contribute to the family economy through subsistence activities, why do they play? Some, such as Bock and Johnson (2004), have argued that some types of play provide children with an opportunity to practice culturally specific and gender-specific complex tasks.

In light of this discussion, using focal follow data from Aka foragers and Ngandu farmers from the Congo Basin, this paper aims to understand the adaptive functions of children’s participation in work and work-themed play. In exploring this topic, we seek to answer two central questions. First, do children’s play and work activities reflect ethno-typical and gender-typical norms within their respective communities? And, second, do children learn complex tasks through work-themed play? Before we outline the steps taken to answer these questions, we will briefly discuss current debates regarding life history and play. We will then review previous studies on play among foragers and other small-scale societies.

## Background

### The Evolution of Childhood

Like other primates, humans have relatively large bodies and invest heavily in a small number of offspring that take a long time to mature. However, some human life history traits do not fit the expected pattern for our clade. Especially for our size, humans have particularly long prereproductive lifespans (Chisholm et al. 1993; Kaplan et al. 2000; Leigh 2001). The several theories that attempt to explain modern human ontogeny and the evolution of human childhood can be roughly divided into two camps. Some argue that selection pressures associated with learning were central to the evolution of childhood; others argue that, though learning is beneficial, other pressures were the drivers of this ontogenetic change (Blurton Jones and Marlowe 2002; Charnov 1993; Charnov and Berrigan 1993; Hawkes 2003). As part of the adult mortality model, those who argue that learning has not been a driving factor in the evolution of childhood hypothesize that extended lifespan and low adult mortality would have delayed maturity, independent of pressures from learning (e.g. Blurton Jones and Marlowe 2002; Hawkes et al. 1998). For example, Hawkes (2003) argues that the insertion of early childhood into modern human ontogeny allows others, especially grandmothers, to care for children directly and indirectly. In doing so, grandmothers increase their inclusive fitness by shouldering some of the cost of their daughter’s current offspring. This allows daughters to reproduce again earlier than if they were solely responsible for the child’s burden of care (Hawkes et al. 1995, 1998; O’Connell et al. 2002). In the adult mortality model, any learning that takes place is beneficial, but not central to the evolution of extended childhood.

On the other hand, Kaplan et al. (2003) argue that childhood is primarily an adaptation for learning. Indeed, the net production of calories in adult humans is much higher than chimpanzees, with some estimates suggesting that hunter-gatherers consume 60% of the calories from calorie-dense, hard-to-extract resources, whereas chimps only consume 2% from such sources (Kaplan and Robson 2002). However, by 15 years of age, foraging children have consumed more than 25% of all the calories they will consume in their lifetimes but have only produced 5% of such calories (Kaplan et al. 2000). Considering this, Kaplan et al. (2003) propose the embodied capital theory, which posits that children trade low productivity and delayed reproduction in early life for high productivity later in life, as well as a longer lifespan. In other words, the pressures for learning to extract calorie-dense resources justifies an extended childhood (Kaplan 1996; Kaplan et al. 2000, 2003; Kaplan and Robson 2002). Research among the Ache of Paraguay found that, though physical strength peaks around the age of 25, Ache hunters reach their peak hunting capabilities in their mid-thirties, lending support to the embodied capital theory (Walker et al. 2002).

As a means of testing these two models, researchers working in other cultural contexts have examined the development of children’s subsistence knowledge and skill and their economic contributions. If children are found to be productive in early life, then the adult mortality model would be supported. The evidence has been mixed. For example, Blurton Jones and Marlowe (2002) demonstrated that more experience did not necessarily improve Hadza forager children’s tuber digging or archery abilities, concluding that the extension of the prereproductive period is not fully explained by selection for greater need to invest in learning. Their study tested people’s knowledge and skill in an artificial “Olympics” contest, but observational studies of foraging children’s daily activities have also found that they can be economically productive in a range of cultural and ecological contexts, and that their productivity may only be limited by growth or ecological constraints, not experience (Bird and Bliege Bird 2005; Bliege Bird and Bird 2002; Blurton Jones et al. 1994; Tucker and Young 2005). Similarly, Kramer (2002) found that children among Mayan maize farmers are net producers for several years before they establish their own families. Maize agriculture, she notes, involves many tasks that do not require great skill or strength. On the other hand, Bock (2002, 2005) has shown that, among part-time forager communities in Botswana, growth and experience additively and independently influence children’s acquisition of skill proficiency. Older children performed better than younger children at chopping wood, cracking mongongo nuts, and pounding grain, even if they had the same level of experience. However, children of the same age with more experience at a task were better skilled. Furthermore, using naturalistic observations of Hadza children, Crittenden et al. (2013) found that both age and personal motivation correlated with Hadza children’s foraging returns.

In sum, evidence suggests children can be productive contributors to subsistence. However, proficiency at essential subsistence skills among humans must be developed in a particular subsistence context, embedded in an environmental setting (Little and Lancy 2016). These factors may influence the degree to which populations rely on easy-to-extract, or hard-to-extract, resources. Unfortunately, few studies have examined whether the learning trajectories of simple versus complex tasks differ. Some have suggested that play—a universal feature of childhood—may function to practice context-specific skills at which children are not yet fully proficient, for reasons of growth, cognitive maturation, or experience. In this study, we aim to further this discussion and examine how children’s time is differentially allocated toward work-themed pretense play (see below) versus productive work among Aka forager and Ngandu farmer children. If children tend to spend more time playing at the complex tasks particular to their subsistence ecology than the simpler ones, then this would support the idea that play is a flexible learning adaptation and provide further evidence for selection on childhood as a period of learning. We develop this prediction more fully in what follows.

### Work and Work-Themed Play in Small-Scale Societies

Play can be defined as “all locomotor activity performed postnatally which appears to an observer to have no obvious immediate benefits for the player, in which motor patterns resembling those used in serious functional contexts may be used in modified terms” (Bekoff and Byers 1981:301; see also Bateson 2014; Beach 1945; Byers and Walker 1995; Fagen 1981). *Pretense play*, defined by Lillard (1993:349) as “the projecting of a supposed situation onto an actual one, in the spirit of fun,” is ubiquitous among humans. Furthermore, the developmental timing of pretense play in children is stable across cultures, suggesting that the emergence of play has a biological and adaptive basis and may make contributions to psychological development (Bornstein 2006; Slaughter and Dombrowski 1989). If play is functional, variations in types of play should provide children with an opportunity to practice behaviors central to survival and reproduction (Bock 2005). In other words, play activities should be ethno- and gender-specific and should prepare children for participation in productive tasks—a facultative design that would be consistent with embodied capital theory. As such, we are specifically interested here in pretense play that involves imitation of work tasks typical of children’s subsistence context, what we will call work-themed pretense play, or simply work-themed play (Boyette 2016a; Fouts et al. 2016).

Previous research conducted among foraging and farming populations does suggest that play makes important contributions to the ways in which children learn subsistence skills, while also serving as a setting for developing cultural competencies, including gender roles and cultural values and norms regarding children’s responsibilities (Boyette 2016a; Dira and Hewlett 2016; Garfield et al. 2016; Gaskins 2000; Gosso et al. 2007; Gray 2009; Imamura 2016; Lew-Levy et al. 2017, 2018; MacDonald 2007; Whiting and Whiting 1975). In terms of subsistence, Bock (2002, 2005) and Bock and Johnson (2004) have hypothesized that if play helps children develop competencies at specific tasks, they will spend less time playing at a task as they get better at performing it. Their research in the Okavango Delta showed that, with age, children do indeed exchange play with work, depending on the labor needs of the household and the complexity of the skill at hand. For example, they found that girls progressively diminished their participation in play grain pounding, stopping around the age of eight, at which point they begin contributing to actual grain pounding. By playing at pounding, girls learn to perform this important task without wasting grain, a costly resource (Bock 2005). Boys, on the other hand, reduced their participation in target games, which stop at around age twelve. The more a family relied on hunted meat for subsistence, the more likely it was for a male child in that family to participate in hunting games. The fact that hunting play continued longer than grain pounding play accords with the complexity of hunting as a subsistence task, requiring extensive ecological knowledge and specialized tools, though not necessarily greater strength (Ohtsuka 1989; Walker et al. 2002).

A prior study of play among the Aka and Ngandu children studied here also found results consistent with a trade-off of play for work as children age. Though specific work-themed play and corresponding work tasks were not examined as they were by Bock and Johnson, Boyette (2016a) reports that age was positively correlated with time spent in work whereas age was negatively correlated with time spent in play. There were also independent effects of both gender and ethnicity on children’s time spent in both work and play. Specifically, consistent with prior research (Draper and Cashdan 1988; Ember and Cunnar 2015; Konner 2005; Munroe et al. 1984; Nag et al. 1978; Rogoff et al. 2003; Whiting and Whiting 1975), Ngandu farmer children worked more than Aka forager children at all ages. Parents in agricultural societies tend to emphasize responsibility and obedience, and task assignment is frequent, whereas forager children are given autonomy over their activities and are rarely coerced. Additionally, girls worked more than boys at all ages independent of ethnicity, consistent with prior cross-cultural research indicating that females assume greater work responsibilities earlier than males (Draper 1975; Munroe et al. 1984; Whiting and Edwards 1973; Whiting and Whiting 1975). Furthermore, Boyette (2016a) found that the Ngandu played significantly more than the Aka, and that the age-dependent decrease in time spent in play was steeper for girls. The last result is again consistent with other research indicating that girls enter the family economy earlier than boys—and consequently give up play at a greater rate. In the current study, we will verify that the results reported for play in Boyette (2016a) hold for work-themed pretense play only, and we also examine how gender, ethnicity, and age relate to individual work and work-themed play activities.

Because gender and ethnicity—at least as it relates to subsistence practices—are intimately tied to work, work-themed play is likely an important venue through which to develop cultural competencies beyond subsistence skills. Indeed, the social aspects of work may be emphasized in the context of work-themed play. For example, among the Kpelle of Liberia, Lancy (1996) found that children participated in blacksmithing play that did not involve accurate mimicry of forging but instead included elements of the social relationships between the master forger and his apprentices (see also Crittenden 2016; Gaskins 2000; Gosso et al. 2007). The emphasis on developing social relationships in work-themed play has been highlighted among foragers as well; a recent review on how forager children learn subsistence skills found that making “play” camps is nearly ubiquitous (Lew-Levy et al. 2017; see also Crittenden 2016; Flannery 1953; Lewis 2002; Lew-Levy et al. 2018; Neuwelt-Truntzer 1981; Shostak 1976; Vanstone 1965). In these camps, children participate in work and work-themed play while emulating adult social interactions, including gender roles (Crittenden 2016; Montgomery 2010; Turnbull 1978) and the social behavior of members of other ethnic groups (Endicott and Endicott 2008; Turnbull 1962). Bock and Johnson (2004) tested the effects of adult subsistence strategy on children’s play and work at the household level. However, the ways in which ethno-typical behaviors at the society level, and the interaction between ethnic groups within a multiethnic community, might influence participation in work and work-themed play have not been explored. The Aka and Ngandu offer an opportunity to examine the commonality of cultural exchange in children’s subsistence play. Though both groups interact and cohabitate for varying periods of time throughout the year, the Aka and Ngandu nonetheless maintain distinct languages, subsistence practices, and cultures (Rupp 2014). Here we seek to understand how interethnic interaction affects children’s activities, a relatively unexplored area of research.

In summary, the present paper will use behavioral observation data to examine how age, gender, ethnicity, and task complexity influence variation in children’s play and work among Aka forager and Ngandu farmer children. Since learning subsistence skills and knowledge requires not only becoming proficient at a set of tasks but also the developmental integration of culture-specific gender and ethnic roles, as we have described, we argue that children’s time allocation toward play activities that are gender-specific, ethnicity-specific, and complex provides support for an adaptive learning function for play and for childhood, in support of the embodied capital theory.

## Hypotheses

Our specific hypotheses are as follows:

1. Independent of ethnicity and gender, age should be negatively correlated with time spent in work-themed play, consistent with a trade-off between play and work as children age, as previously established for the Aka and Ngandu by Boyette (2016a).
2. Aka and Ngandu children will spend more time imitating complex activities than simple activities in play, consistent with the proposal that work-themed play constitutes investment in embodied capital.
3. Aka and Ngandu children will participate in work and work-themed play activities that reflect ethno-typical and gender-typical behaviors in their respective societies, consistent with the developmental integration of the cultural contexts of subsistence.

## Methods

### Study Populations

The children included in this study are from farming and foraging communities in the northwestern region of the Congo Basin, in the Central African Republic. Though both societies share the same ecological context, the Aka and Ngandu nonetheless maintain distinct cultural practices and subsistence strategies. The Aka live in camps of approximately fifteen related nuclear families who forage and share food with each other (Hewlett 1991). *Koko* (*Gnetum africanum*) is an important gathered plant, is highly nutritious, and is used as medicine and food throughout the region. Net hunting, spear hunting for large mammals, and bow or crossbow hunting for monkeys and large birds is the main source of protein for the Aka (Thomas and Bahuchet 1991). Honey is also a valued seasonal resource (Bahuchet 1988). The Aka maintain small gardens in which they grow manioc (cassava) and bananas, two important sources of carbohydrates. Despite the presence of these gardens, foraged and hunted foods remain an important part of Aka diet and identity. Like other Congo Basin foragers, the Aka maintain trade relations with nearby farmers. In the region, these are primarily of Ngandu ethnicity. The Ngandu are slash-and-burn agriculturalists whose main crops include manioc, corn, plantain, oil palm, peanuts, and taro (Hewlett et al. 2011). Though some domestic livestock is kept, men also hunt and trap wild game in the forest (Thomas and Bahuchet 1991). Surplus is often sold from house to house, or in the central market. Depending on the current market and governmental situation, coffee crops are also sold to the Central African government for international trade. The Aka frequently work in Ngandu gardens; sell koko and leaves needed for making *chiquan*, a traditional loaf made of manioc; and participate in gun or crossbow hunting for cash.

### Aka and Ngandu Childhood

Like other foragers, and unlike the Ngandu, the Aka are highly egalitarian, share food widely, and value cooperative autonomy. In comparison, the Ngandu place special cultural importance on the values of hierarchy, competition, authority, wealth, and prestige. These differing cultural values lead to differing learning ecologies (Hewlett et al. 2011; Boyette and Hewlett 2017). Both Aka and Ngandu children spend an increasing amount of time in playgroups as they get older (Boyette 2016a, b; Hewlett et al. 2011). Within these groups, the Ngandu played competitive games six times more often than the Aka and are two times more likely to play rough-and-tumble games than the Aka, probably because of the importance of competition in Ngandu culture (Boyette 2016a). On the other hand, Aka games, such as *ezambi*, which involves swinging on a liana, foster cooperation with each other and engagement with the forest.

Like other small-scale agriculturalists (e.g., Whiting and Whiting 1975), Ngandu girls and boys inhabited different learning environments; Ngandu children spend more time with peers of their own age and gender than Aka children (Hewlett and Roulette 2014). These learning environments are often mediated by chore assignment, and, combined with a rigid division of labor in adulthood, influence the development of gendered behaviors (Boyette 2016a; Munroe et al. 1984; Whiting and Whiting 1975). Because there are fewer same-aged peers in Aka camps, Aka children are more likely to play in multi-aged and mixed-gender groups. Furthermore, owing to the importance of autonomy, Aka children are rarely assigned chores and are not punished for noncompliance with these assignments (Boyette and Hewlett 2017). Finally, Aka adults do not maintain a rigid division of labor, as both men and women participate in net hunting, harvesting, and other opportunistic foraging activities (Thomas and Bahuchet 1991). For many farming adolescents, including the Ngandu, failure to assist in the household economy could lead to physical punishment and social sanctions. Though Aka adolescents do help their families with subsistence and childcare tasks, they are not forced to do so, and they do not experience social sanctions if they choose not to (Hewlett and Hewlett 2012).

Aka and Ngandu children have the opportunity to observe the other group’s subsistence activities during much of the year. The Aka families included in the study live in close proximity to the Ngandu village from three to nine months of the year, when they often perform labor for the Ngandu. Additionally, each year during caterpillar season, the Ngandu live in the forest from a few weeks to two or three months at a time at their own camps, in proximity to Aka caterpillar camps. Furthermore, Ngandu men often hire Aka men to join hunting parties, and adolescents from both groups are often included on these excursions. Today, shotgun hunting is the standard, but in the past the Ngandu used spears like the Aka did to hunt and would even join net hunts. During the field work period, one Ngandu elder still left his home every day carrying his spear. Thus, shared cultural knowledge of even some of the most ethnicity-specific subsistence practices does exist, though it is not as widespread as it once was.

### Data Collection

The time allocation data for individual Aka and Ngandu children analyzed here were collected by the second author between March and September 2010. A census of eight forest-dwelling Aka communities and 18 Ngandu families was conducted. At the time of data collection, Aka children did not attend school. Though school was available to Ngandu children, it was often closed, so children’s attendance was sporadic. Nonetheless, Ngandu children were observed outside of school hours. 50 Aka children and 48 Ngandu children were selected at random based on age and gender. Verbal consent was obtained from parent and child before the observation period began. If either parent or child did not provide consent, another child matching in age, ethnicity, and gender was chosen at random from the census. One Ngandu father and one Aka child refused to participate.

The age of Aka children was estimated by the second author and his Ngandu field assistant, who grew up around the Aka and thus knew many of the families surveyed. When these estimates disagreed by more than two years, parents were asked to rank their children based on birth order. By comparing the child in question to ones of known age, an estimated age was derived. The Ngandu knew the ages of their children in years. Ages were collapsed into three categories based on life history stages, as well as Aka and Ngandu conceptions of developmental stages. Table 1 shows the sample breakdown by age category, gender, and ethnicity.

**Table 1:** Age categories used in the analysis, and number of participants based on gender, age, and ethnicity (based on Boyette 2013)

Age (yrs)	Developmental stage	Local name of developmental stage		Participants				Total
				Aka		Ngandu		
		Aka (DiAka)	Ngandu (Songo)	Girls	Boys	Girls	Boys	
4–6	Early childhood	*mona*	*molengue*	8	8	8	7	31
7–12	Middle childhood/Juvenility	*mona ngondo* (f) /*bokala* (m)	*kete masika wali* (f) / *koli* (m)	10	10	11	10	41
13–16	Adolescence	*ngondo* (f) / *bokala* (m)	*masika wali* (f) */ koli* (m)	8	6	5	7	26
Total				26	24	24	24	98

Data were collected using focal follows and interval sampling (Altmann 1974). Children were randomly assigned three 2-h sample periods over a series of days, such that each child was observed during one morning, midday, and afternoon period. All observations were conducted between 6 am and 6 pm. If a child was not available for observation during a particular time slot, the observation was rescheduled or omitted based on circumstance. Children were observed for 45 min followed by a 15-min break. Behaviors were coded using a 30-s-observe/30-s-record procedure. Observations when children were out of sight were omitted. Omitted data resulted in a range of observations per child from 46 to 322, for a total of 22,896 observations.

The behavioral coding scheme was modeled on similar studies of social learning and play (Bock 2002; Gaskins 2000) and adjusted to the field site based on fieldwork conducted in and outside the village of Bagandou in July through November 2008. Children were coded as participating in work-themed play or work. For the purposes of this study, work-themed play is defined as imitation of work without productive ends (Boyette 2016a). Conversely, work is defined as any activities that contribute to personal and/or family subsistence (Munroe et al. 1984). All children recorded as performing work-themed play or work were also coded on the specific type of activity performed. Definitions for these activities, as well as examples of behaviors included in these categories, are provided in Table 2.

**Table 2:** Description of activities coded and examples

Activity	Description	Example
Gathering	Harvesting fruit, berries, *koko* leaves, nuts, caterpillars, and digging for tubers.	Work: Climbing a tree and bringing down *koko* bundles.
		Play: Digging as if to acquire tubers where none grow.
Net hunting	Manufacture and use of specialized nets from *mokoso* vines, which are laboriously processed by men. While women and children beat bushes to scare out prey towards nets, the men guard nets set up in a specific area of the forest, ready to capture and kill those species which are caught in them.	Work: Participating as beaters or pursuing game during a net hunt
		Play: Using an old hunting or fishing net to capture a chicken, which is not eaten.
Spear hunting	Following an animal trail, and being readily positioned and strong enough to thrust the spear into the animal.	Work: Pursuing forest mice or lizards which are eaten.
		Play: Spearing fruit or a moving target.
Trapping and Snaring	Locating an animal trail, and setting a trigger trap to which a snare is set. The trap must be set just-so, or injury from the trigger snapping unexpectedly can be very painful.	Work: Placing a trap in the forest along an observed animal trail.
		Play: Making traps in camp or around the house in the village.
Other hunting	All other hunting activities, including crossbow or sling shot hunting.	Play: This was typically play with sling shots and not a serious way to hunt for children.
Honey harvesting	Finding honey from stinging or stingless honey bees in trees, fallen branches and in the ground. Also includes climbing, chopping, and filling containers with honey.	Work: Participation in the cooperative labor of honey extraction.
		Play: building a small honey basket; climbing a tree with no honey but bringing a leaf bundle to “smoke out” the bees.
Food preparation	Gutting, peeling, cutting, husking, boiling, grilling, grating, or frying. Also includes fetching water and harvesting firewood.	Work: Preparing food to eat.
		Play: “Cooking” inedible leaves or grass in a sardine can (over a real or pretend fire).
House construction	Aka; building round huts out of large, waterproof leaves readily available in the environment. Ngandu; building mud huts which are covered in palm leaf tiling.	Work: Helping to prepare roofing leaves.
		Play: Building miniature houses of toy or child size.
Fishing	Bail fishing, common among the Aka; fishing with traps, common among the Ngandu; and fishing with hooks.	Work: Participation in bail or pole fishing.
		Play: Imitation of bail fishing in camp after a rainstorm.
Commerce	Involves selling items such as palm wine, meat, fish, goods to neighbors, door to door, or at the market.	Work: Running the family market stand or selling goods on foot through the village.
		Play: “Selling” goods to other children for imitation currency or trade goods.
Gardening	Clearing land, planting, weeding and harvesting of manioc, maize, and bananas, and other, less common gardened plants.	Work: Harvesting; planting manioc stocks.
		Play: Clearing weeds from camp or house spaces using motions typical of garden work.
Other miscellaneous work activities	All other work activities, including laundry, making or maintaining tools or gathering forest cords for various uses (e.g., rope or basket making)	Work: Preparing vines for basket weaving.
		Play: Making cord from inappropriate vine in imitation of rope making.

### Data Analysis

#### Participation in Work-Themed Play across Development

Hypothesis 1 was tested using negative binomial regression modeling following Boyette (2016a). We built two models. Count of observations spent in work-themed play per child was the dependent variable in both models. Model 1 included the main effect of age, gender, and ethnicity as independent variables. In addition to the main effects, Model 2 included all two-way interactions. In both models, the natural log of the total number of observations per child was included as an offset variable to account for variation in total intervals of observation per child (Long and Freese 2006).

#### Participation in Simple Versus Complex Activities

In order to investigate how task complexity influenced children’s activity choice within each ethnic group, a comparison of the proportion of observations each child spent in work versus work-themed play for each activity type was conducted (e.g., gathering vs. play-gathering) using Wilcoxon signed-rank tests. These tests were conducted on the Aka and Ngandu samples separately, to allow for a meaningful comparison of culture-specific activities. Children of all ages were included in the analysis because tests by age category would have had insufficient cell sizes for statistically meaningful analysis of each activity type.

We also conducted two Wilcoxon signed-rank tests on activity categories defined as “simple” versus “complex” for both the Aka and Ngandu. Based on our ethnographic observations, as well as unstructured interviews on the difficulty of a variety of skills, we have categorized the activities as follows: simple activities include miscellaneous work, food preparation, fishing, commerce, gathering, and gardening. Among these activities, miscellaneous work includes such chores as running errands or doing laundry; commerce includes selling goods (typically fruits, vegetables, meat, salt, sugar, coffee, soap, or other small goods) door-to-door or at family-owned stands along the roadside. Complex activities include net hunting, trapping, spear hunting, and other types of hunting. Age-related trends for participation in complex and simple activities were visually investigated to determine how work and work-themed play trade-off within these two categories.

Apart from ethnographic justifications, there are theoretical reasons to divide these categories in this manner. For example, the resources included in simple activities are fixed in space (fish cannot leave their pond; cassava cannot walk away). Also, the Aka and Ngandu do not usually use specialized tools to complete them (with the exception of graters and pounders used to process manioc leaves). Finally, these simple activities can be readily observed because inexperienced children cannot interfere with them (for example, by scaring off prey). Though some gathered resources can be complex to find, gather, and process, this is rarely the case for the wild foods consumed by Congo Basin farmers and foragers. Indeed, wild sweet potato (*mela*) usually grows in abandoned forest camps, and thus locating these resources is straightforward (see also Tucker and Young 2005). Furthermore, unlike the mongongo nuts harvested in the Kalahari (Blurton Jones et al. 1994), various nut varieties are easy to find, and are not difficult to husk and shell; indeed, inexperienced anthropologists have done so with success (see Boesch et al. 2017 for description of processing different Congo Basin nut varieties).

Activities in the complex category were defined as such because they require intimate knowledge of the environment (to find animal trails and tracks), involve specialized tool use, and, in the case of trapping, require complex tool construction. These activities are not usually performed in the company of children and thus are not readily observed. We do not define complexity based on difficulty in terms of strength; gardening and gathering can require immense upper body strength for successful completion. Also, as shown by Ohtsuka (1989) and Walker et al. (2002), strength is not necessarily correlated with greater hunting success, highlighting the limitations of strength as a good measure for task complexity.

Finally, exploring simple and complex activities as categories provides us with a more realistic understanding of the ways in which these skills are learned since the knowledge learned in one activity readily applies to many others. Indeed, learning the ecological knowledge necessary for finding animals in the context of trapping is also applicable to hunting with a net, or hunting with a spear. Similarly, learning to harvest leaves for house construction is also applicable to gardening and gathering. Thus, we examined these categories as a whole, instead of in parts.

#### Participation in Ethno-Typical and Gender-Typical Activities

Finally, Hypothesis 3 was tested using two sets of Mann-Whitney U tests on each work and work-themed play type. In the first set, ethnicity was used as the grouping variable to help us understand how membership in a cultural group influences children’s participation in work and work-themed play. In order to understand how gender influenced children’s participation in work and work-themed play, the second set were conducted for the Aka and Ngandu separately, using gender as the grouping variable. In each set, the proportion of observations spent in a work or work-themed play activity was calculated by dividing the frequency of work and work-themed play by the total number of observations for each child. Again, children of all ages were lumped together because cell sizes were too small for statistical tests of individual activity frequencies by age category.

All analyses were conducted using IBM SPSS 22. All values were considered statistically significant at or below *p* = 0.05.

## Results

### Descriptive Statistics

The mean proportions of observations spent in work and work-themed play by activity category are presented in Table 3. The Ngandu worked more and participated in more work-themed play than the Aka. Independent of ethnicity, girls worked more than boys. Ngandu girls participated in more work-themed play than boys, whereas the reverse was true for the Aka. Overall, participation in gathering was the most common work activity for Aka boys and girls, whereas participation in food preparation was the most common work activity for Ngandu girls and boys. Food preparation play was the most common play for Aka girls, whereas playing at net hunting was the most common play for Aka boys. Playing at food preparation was the most common play activity for Ngandu girls, whereas playing at trapping was the most common play activity for Ngandu boys. Because honey harvesting was never observed, and honey harvesting play was only observed once, it is included in the negative binomial regressions (immediately below) but excluded in all other analyses.

**Table 3:** Mean percent of observations spent in work and work-themed play per activity, gender, and ethnicity

Activity		Aka (mean % of obs)			Ngandu (mean % of obs)		
		Boys	Girls	All	Boys	Girls	All
Gathering	Work	4.08	11.30	7.98	0.53	0.95	0.74
	Play	0.21	0.89	0.58	0.02	0.33	0.18
Net hunting	Work	0.95	0.77	0.85	0	0	0
	Play	1.99	0.47	1.17	0	0	0
Honey	Work	0	0	0	0	0	0
	Play	0.02	0	0.01	0	0	0
Spear hunting	Work	0	0	0	0	0	0
	Play	0.67	0	0.31	0	0	0
Trapping	Work	0	0	0	2.67	0	1.34
	Play	0.06	0	0.03	2.91	0	1.45
Other hunting	Work	0.38	0	0.17	0.34	0	0.17
	Play	1.15	0.21	0.64	0.77	0.08	0.43
Food preparation	Work	3.85	8.32	6.26	6.32	13.87	10.10
	Play	0.27	1.01	0.67	0.14	3.49	1.81
House construction	Work	0.02	0.09	0.06	1.30	0	0.65
	Play	0	0	0	0	0.23	0.11
Fishing	Work	0	0.59	0.32	0.19	0	0.09
	Play	0	0.04	0.02	0	0.03	0.02
Commerce	Work	0.05	0.03	0.04	3.57	3.12	3.35
	Play	0.02	0.16	0.09	0.46	0.62	0.54
Gardening	Work	0	0.01	0.01	0.85	0.45	0.65
	Play	0	0	0	0.03	0.02	0.02
Miscellaneous activities	Work	1.03	1.75	1.42	5.34	12.25	8.79
	Play	0.86	0.80	0.83	0.05	0.38	0.22
Total	Work	10.35	22.86	17.1	21.11	30.65	25.88
	Play	5.24	3.58	4.34	4.38	5.18	4.78

### Age and Participation in Work and Work-Themed Play

**Fig. 1:**
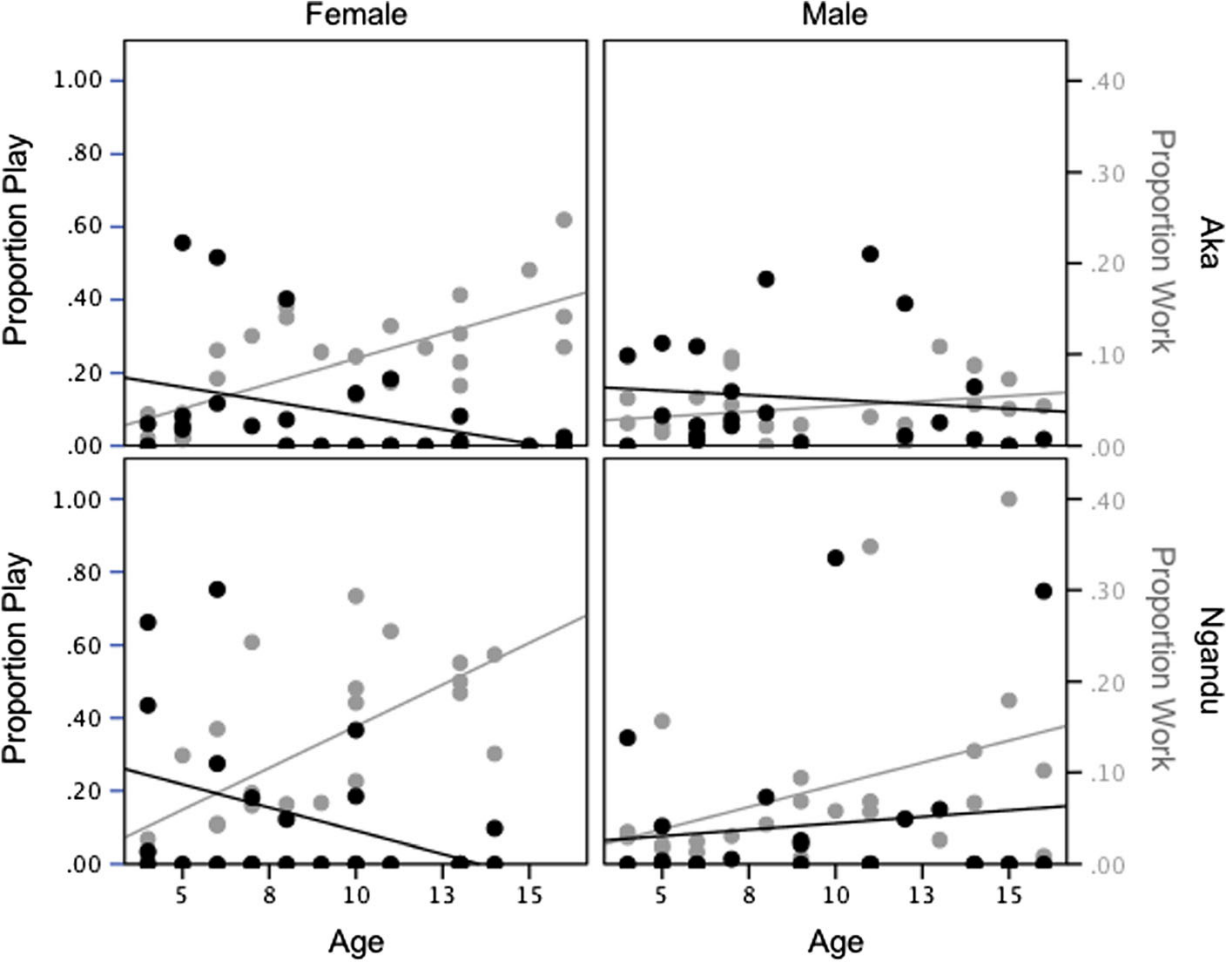
Proportion of observations spent in work and work-themed play by age, sex, and ethnicity. Based on focal follows conducted in 2010 of 50 Aka and 48 Ngandu children ranging in age from 4 to 16 (Boyette 2016a)

Hypothesis 1 was tested using negative binomial regression modeling. Model 1, including only the main effects, was significant (Model LR χ^2^_(3)_ = 12.21, *p* = 0.007). Only age had a significant, and negative effect on participation in work-themed play (B = −0.11, *p* = 0.001), indicating that children reduced their participation in work-themed play with age. Model 2, including main effects and two-way interactions, was also significant (Model LR χ^2^_(6)_ = 32.39, *p* < 0.001). Only gender and the interaction of gender and age were significant. Gender was a significant predictor for participation in work-themed play (B = 2.20, *p* = 0.001), indicating that boys participated in more work-themed play than girls. The interaction between age and gender was also significant (B = −0.26, *p* < 0.001), indicating that girls decreased their participation in work-themed play at a quicker rate than boys. Figure 1 plots the linear relationship between age versus play/work by ethnicity and gender.

### Participation in Simple Versus Complex Activities

Hypothesis 2 was tested using Wilcoxon signed-rank tests comparing frequencies of work and work-themed play for each activity. Results are presented in Table 4. These results indicate that Aka and Ngandu children spent significantly more time gathering than playing at gathering (Aka: *p* < 0.001, Ngandu: *p* = 0.05), significantly more time participating in food preparation than they did at playing the same activity (Aka: *p* < 0.001, Ngandu: *p* < 0.001) and significantly more time doing other, miscellaneous activities than they did at playing at these same activities (Aka; *p* = 0.032, Ngandu: *p* < 0.001). Ngandu children also spent significantly more time participating in commerce than they did playing at commerce (*p* = 0.007) and significantly more time participating in gardening than they did playing at gardening (*p* = 0.011).

**Table 4:** Results of Wilcoxon signed-rank tests of whether each activity was more frequently seen as work or play for each ethnicity

Activity	Aka		Ngandu	
	*T*	*z*	*T*	*z*
Gathering	52.5	−4.99***	52.5	−1.96*
Food preparation	75.0	−4.71***	129.5	−4.03***
House construction	0	−1.34	2.0	−0.54
Fishing	1.0	−0.45	1.0	−1.07
Commerce	6.0	−0.41	50.0	−2.68**
Gardening	0	−1.00	1.0	−2.55*
Miscellaneous activities	280.0	−2.15*	10.0	−5.52***
All “simple” activities	38.0	−5.57***	69.0	−5.32***
Net hunting	21.0	0.66	0	0
Spear hunting	0	1.83	0	0
Trapping	0	1.34	16.0	0.28
Other hunting	16.0	1.80	8.0	1.40
All “complex” activities	56.0	1.57	44	0.91

Negative *z* values indicate that the mean negative rank was larger and thus that work occurred more frequently than play. Positive *z* values indicate the opposite

*p≤0.05; **p≤0.01; ***p≤0.001

**Fig. 2:**
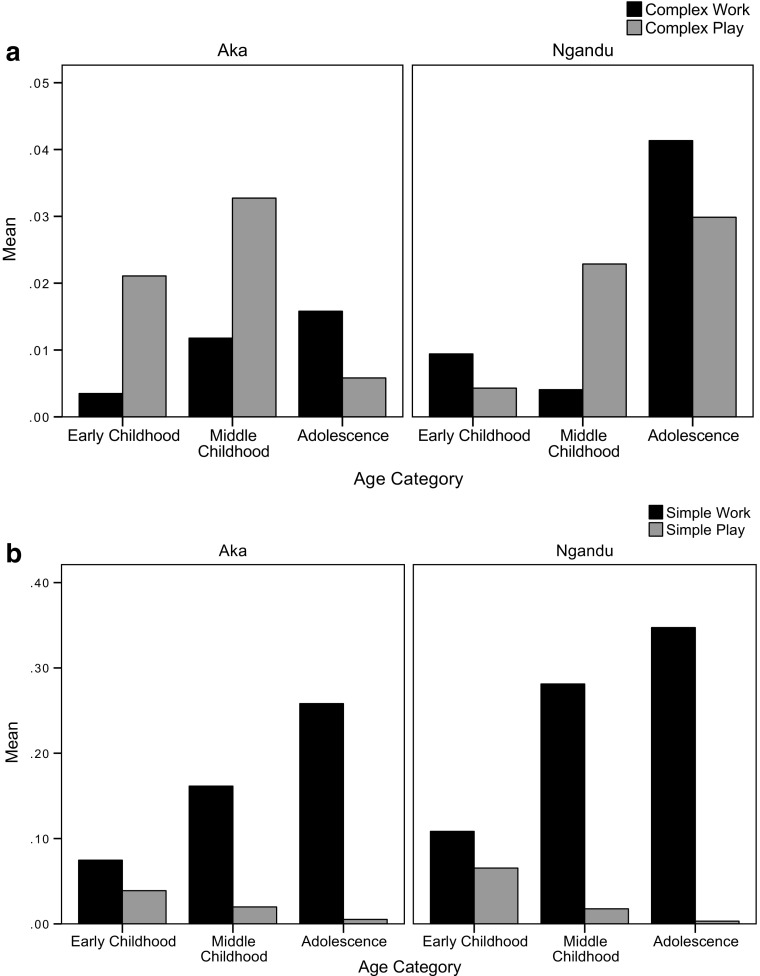
Children’s participation in (a) complex and (b) simple work and work-themed play by age category. Simple activities include gathering, food preparation, house construction, fishing, commerce, gardening, and other miscellaneous work activities. Complex activities include trapping, net hunting, spear hunting, and other forms of hunting. Based on focal follows conducted in 2010 of 50 Aka and 48 Ngandu children ranging in age from 4 to 16 (Boyette 2016a)

When comparing the aggregated simple and complex activity categories, we find that for both the Aka and Ngandu, children work more than they play at simple activities (Aka: *p* < 0.001, Ngandu: *p* < 0.001). Though both Aka and Ngandu children played more than they worked at complex activities, the differences were not statistically significant. Figure 2 shows the relationships between age category and participation in work and work themed play. For both the Aka and Ngandu, children play more at complex activities until adolescence, at which point a major increase in participation in work when compared to play is apparent (Fig. 2a). On the other hand, for simple activities, though Aka and Ngandu children play less and work more overall as they age, they work more at every stage of childhood than they play (Fig. 2b).

### Participation in Ethno- and Gender-Typical Activities

Hypothesis 3 was tested using Mann-Whitney tests. Results for ethnicity are presented in Table 5. These results indicate that the Aka spent significantly more time gathering (*p* < 0.001) and play-gathering (*p* = 0.04) than the Ngandu. The Aka also spent significantly more time playing at net (*p* = 0.007) and spear hunting (*p* = 0.047) than the Ngandu. The Ngandu, on the other hand, spent significantly more time participating in trapping than the Aka (*p* = 0.038). Ngandu children spent significantly more time participating in commerce (*p* < 0.001), gardening (*p* = 0.011), and miscellaneous work activities (*p* < 0.001) than their Aka counterparts, whereas Aka children spent significantly more time playing at miscellaneous work activities than the Ngandu (*p* = 0.002).

**Table 5:** Results of Mann-Whitney tests of difference in frequency of work and work-themed play by ethnic group

Activity		U	*z*
Gathering	Work	407.5***	−5.85
	Play	993.0*	−2.05
Net hunting	Work	1128.0	−1.72
	Play	1032.0**	−2.67
Spear hunting	Work	1200.0	0
	Play	1104.0*	−1.99
Trapping	Work	1100.0*	2.07
	Play	1144.0	0.96
Other hunting	Work	1179.0	−0.39
	Play	1154.0	−0.48
Food preparation	Work	1063.0	0.98
	Play	1188.0	0.12
House construction	Work	1197.0	0.06
	Play	1175.0	1.02
Fishing	Work	1175.0	0.60
	Play	1199.5	0.02
Commerce	Work	757.0***	4.24
	Play	1142.0	0.87
Gardening	Work	1020.0*	2.55
	Play	1150.0	1.45
Miscellaneous activities	Work	505.0***	4.95
	Play	857.5**	−3.05

Negative *z* values indicate that the mean rank was larger for the Aka, and thus the Aka participated in the activity more than the Ngandu did. Positive *z* values indicate the opposite

*p≤0.05; **p≤0.01; ***p≤0.001

Results for gender are presented in Table 6. Aka girls spent significantly more time gathering (*p* = 0.02), participating in food preparation (*p* = 0.017), and participating in other, miscellaneous work activities (*p* = 0.018) than Aka boys. Aka boys were significantly more likely to play at spear hunting (*p* = 0.025) and other hunting (*p* = 0.018) than Aka girls. The results also indicate that Ngandu boys were significantly more likely to participate in trapping (*p* = 0.039) and play-trapping (*p* = 0.039) than Ngandu girls, whereas Ngandu girls were significantly more likely to participate in food preparation (*p* = 0.001) and in other, miscellaneous work activities (*p* = 0.028), as well as playing at food preparation (*p* = 0.009) and at other, miscellaneous work activities (*p* = 0.048) than Ngandu boys.

**Table 6:** Results of Mann-Whitney tests of difference in frequency of work and work-themed play by gender for each ethnic group

Activity		Aka		Ngandu	
		U	*z*	U	*z*
Gathering	Work	191.0*	−2.33	282.0	0.15
	Play	279.5	−0.74	239.0	−1.76
Net hunting	Work	302.0	−0.4	288.0	0
	Play	265.0	1.47	288.0	0
Spear hunting	Work	310.5	0	288.0	0
	Play	256.5*	2.23	288.0	0
Trapping	Work	310.5	0	240.0*	2.07
	Play	283.5	1.55	240.0*	2.07
Other hunting	Work	270.0	1.92	264.0	1.43
	Play	225.5*	2.37	237.0	1.62
Food preparation	Work	188.5*	−2.38	126.0***	−3.34
	Play	292.5	−0.5	202.0**	−2.6
House construction	Work	309.0	0.09	264.0	1.43
	Play	310.5	0	276.0	−1
Fishing	Work	299.0	−0.92	264.0	1.43
	Play	299.0	−0.92	276.0	−1
Commerce	Work	296.0	0.69	254.0	−0.78
	Play	301.0	−0.45	277.0	−0.43
Gardening	Work	299.0	−0.92	269.5	−0.59
	Play	310.5	0	287.5	0.03
Miscellaneous activities	Work	190.0*	−2.36	181.5*	−2.2
	Play	298.5	0.26	229.0*	−1.98

Negative *z* values indicate that the mean rank was larger for girls, and thus girls participated in the activity more than boys did. Positive *z* values indicate the opposite

*p≤0.05; **p≤0.01; ***p≤0.001

## Discussion

Understanding the role of play in the acquisition of subsistence skills and the development of ethno-typical and gender-typical behaviors can make important contributions to our understanding of the role of learning in the evolution of childhood. In this paper, we examined work and work-themed pretense play among Aka forager and Ngandu farmer children of the Congo Basin and tested three hypotheses: First, time spent in work-themed play will be negatively correlated with age, independent of ethnicity or gender; Second, children will play more than they work at complex activities; and third, children will participate in ethno-typical and gender-typical activities across childhood. We found support for the first and third hypothesis, and although we did not find evidence supporting our second hypothesis, the results prompt a rethinking of the distinction between work and play in the acquisition of subsistence knowledge and skill, and they add important insights into the role of childhood in learning and the evolution of human life history. We will place each primary result in the context of current research and then discuss overall implications for the evolution of human childhood.

### Age and Participation in Work-Themed Play

Boyette (2016a) reported that, among the same sample of Aka and Ngandu children studied here, the older a child was, the more likely they were to work and less likely they were to play in general. Not surprisingly, we have shown that this holds true for work-themed play activities independent of other play types. This continues to be consistent with the idea that play is a venue for learning adaptive skills and knowledge, and as such it is also consistent with childhood having evolved to support learning complex subsistence tasks, as predicted by the embodied capital theory. Children practice skills and knowledge specifically related to subsistence through play, and as they acquire competence, they apply it to productive work (Bock 2005).

### Complex Versus Simple Activities

Bock and Johnson (2004) proposed that the subsistence demands of the family economy and the complexity of the task in question influence how children will spend their time. Based on these predictions, we hypothesized that children practice particularly complex, culturally salient subsistence activities through work-themed play and will therefore play at those activities more than work at them. The results presented here do not support this hypothesis. We did not find a statistically significant difference in frequency of work-themed play versus work at complex activities (i.e., hunting). Instead of work-themed play at complex activities occurring significantly more than work at those activities, work occurred significantly more than work-themed play at simple activities. For example, Aka children worked more than they played at gathering, while Ngandu children spent more time gardening than play gardening. As mentioned, both the Aka and Ngandu rely extensively on plant food, either gathered or grown, for subsistence (Marlowe 2007; Thomas and Bahuchet 1991). Farmed and gathered resources, though requiring skill and strength to acquire, have a predictable encounter rate. In addition to harvesting, children from both cultures participated in cooking and miscellaneous activities more than they played at the same activities. Once again, these activities usually take place in and around the camp or homestead, where children can readily observe and participate at will. Similarly, Ngandu children spent more time participating in commerce than playing at this same task, an activity which requires complex social skills but which can easily be learned while doing. Thus, for these activities, “on-the-job training” is most appropriate as children can learn while contributing to the household economy.

On the other hand, both Aka and Ngandu children spent less time participating in complex work—in this case, hunting—than they did playing at complex work, though the difference was not statistically significant. Hunted meat, an important resource for both cultures, is also an unpredictable one (Roscoe 2006). Hunting requires more refined physical and mental skills, including ethno-ecological knowledge, track and sign interpretation, strength, precision, stalking, and tool use (MacDonald 2007). The results presented above suggest that, though participation in these complex activities is rare overall across childhood, children do play at them more than, or as often as, they participate in them as work. Furthermore, although statistical analysis of developmental trends was not possible due to small sample sizes, when we examined the age trends in complex versus simple work-themed play and work, a striking spike in participation in complex work-themed play in middle childhood for both the Aka and Ngandu was apparent, highlighting the importance of middle childhood in acquiring culturally relevant skills. Indeed, across cultures, it would seem that middle childhood is an important time for learning complex skills in playgroups (Lancy and Grove 2011). For example, MacDonald (2007) and Lew-Levy et al. (2017) found the importance of learning in middle childhood to be particularly relevant to hunting skills. In accordance with Bock and Johnson (2004), our analysis reveals that by adolescence, children spent more time “learning by doing,” including at complex activities; participation in hunting as opposed to play hunting takes up proportionally more time throughout adolescence.

### Participation in Ethno- and Gender-Typical Activities

#### Ethnicity

Our results also show that the Ngandu were more likely to participate in village activities (i.e., farming and commerce), miscellaneous activities, and trapping, whereas the Aka were more likely to participate in gathering play and work, and playing at spear hunting and net hunting. These findings may highlight the ways in which cultural beliefs about ethno-typical behaviors, rather than the everyday practice of activities, influence children’s activity choice. Indeed, though Aka adults do trap regularly, this activity was likely brought into the region during the Bantu expansion, and it continues to be thought of as foreign. For example, one Aka informant told us, while setting up a trap himself, that “trapping comes from the farmers, not from us.” On the other hand, in recent years, net hunting has decreased in the surveyed Aka community, with only one family observed by the second author to regularly net hunt during his fieldwork over the course of four years. Despite this, Aka children still played at net hunting, whereas no Ngandu children were observed doing so, though they certainly know about the practice. Similarly, among the San, where hunting by adults has diminished, Imamura also found that hunting play continued, arguing that “children’s hunting play might be regarded as containing a collective memory of traditional hunting activities” (2016:184).

Even in village camps, where Aka participation in gardening and commerce is common, the Aka continue to pride themselves on their orientation toward the forest. Thus, it may be that beliefs about culturally appropriate behaviors can explain why Aka children were less likely to participate in gardening, commerce, and trapping; despite waning in everyday occurrence, net hunting continues to be culturally salient for the Aka, and the opposite may be true for gardening and commerce. Our findings, and those of Imamura, support the hypothesis that children will participate in ethno-typical play and work activities despite extended contact and cultural exchange with other-cultured individuals. Furthermore, though we are certainly not the first to find that children’s play imitates the activities of adults within their culture (Lancy 2008), to our knowledge, no studies have specifically addressed the fact that ethno-typical work-themed play persists in multiethnic communities.

A handful of other studies have also found evidence that children will preferentially imitate adults who share their ethnicity. For example, in an experimental study of Euro-American infants and preschoolers, children were found to copy the actions of those who are native speakers in the child’s language more than non-native speakers (Buttelmann et al. 2013; Over and Carpenter 2013). Henrich and McElreath (2003) have proposed that ethnic markers arose so that individuals could identify members within their ethnic group and coordinate behaviors with regard to subsistence practices, marriage, inheritance, and conflict resolution. These arbitrary symbols allow individuals of different ethnicities to live in close proximity while retaining cultural variation. In order to learn these coordinated behaviors, children are biased to learn from individuals who share their ethnic markers. Though we have not explicitly tested for this bias throughout this paper, our results do contribute supporting evidence for ethnic bias among Congo Basin farmers and foragers.

It is imperative to note that while this research suggests ethnic bias in children’s learning supports the acquisition of the skills and knowledge most relevant to a child’s cultural context (e.g., as indicated by ethnic markers), ethnic boundaries are fluid and obviously permeable to interethnic cultural learning. For example, Endicott and Endicott (2008) observed Batek children in Malaysia imitating migrant Indian and Chinese shopkeepers. Similarly, the second author has observed Aka children playing at the exchange of koko leaves for market goods—an activity that would have been coded as commerce play, involving both Ngandu and Aka roles. Notably, these particular children lived in a camp where the adults collected more koko for the Ngandu merchants than in other camps visited during fieldwork, and Ngandu *koko walis* (koko women) were frequently living in the forest camp for days or weeks. The Aka in this particular camp, including the children, also spoke Sango, the Central African national language spoken commonly by the Ngandu, more than elsewhere. Thus, although anecdotal at this point, evidence suggests human developmental psychology is sensitive to markers of culture change as well as ethnic markers, and work-themed pretense play is likely a useful arena in which to study both.

#### Gender

Boyette (2016a) found that Aka and Ngandu girls worked more than boys of each group at all ages, and that girls, independent of ethnicity, decreased time devoted to play in general at a greater rate than boys. Here, we confirmed that girls decreased their time spent in work-themed play at a greater rate than boys, but, in addition, the types of work and work-themed play in which children participated were also patterned by gender, consistent with the early emergence of a gendered division of labor. We found that gathering, food preparation, and miscellaneous work were performed more frequently by girls than by boys, whereas boys spent more time in hunting and play hunting than girls. These findings reflect adult gender-typical behaviors among both the Aka and Ngandu and are similar to those found throughout the Congo Basin (Gallois et al. 2015; Hewlett and Cavalli-Sforza 1986). Thus, our findings support the hypothesis that children engage in gender-typical activities across childhood.

Chore assignment is usually used to explain why girls’ and boys’ participation in work differs (Konner 2005; Munroe et al. 1984; Whiting and Whiting 1975). However, though not explicitly measured and tested in the present paper, various authors have noted that, among Congo Basin foragers in general and the Aka in particular, chore assignment is less common than among subsistence farming populations, such as the Ngandu (Berry et al. 1986; Boyette 2016a; Boyette and Hewlett 2017; Hewlett and Cavalli-Sforza 1986; Morelli 1997). Furthermore, when chore assignment does occur, children rarely experience repercussions when they refuse to comply (Boyette and Hewlett 2017). In the absence of chore assignment, Draper (1975) proposed the theory of identification to explain why hunter-gatherer children participated in gendered activities independently of direct parental instruction. Similar to the ethnic bias described above, through identification, children choose same-gender adults as models. Children mimic these models and monitor their own actions in reference to the models’ actions. Various authors have also noted this gender bias among foraging societies (Draper 1975; Endicott and Endicott 2008; Flannery 1953; Fouts and Hallam 2013; Fouts and Lamb 2009; Gallois et al. 2015; Wallace and Hoebel 1952). Thus, the present paper lends support to the hypothesis that children identify with models who are most like them and suggests that participation in work and work-themed play are two ways in which children practice gender-typical behaviors. It is also consistent with the large ethnographic literature indicating that girls universally work more and play less than boys, independent of subsistence strategy, starting as early as middle childhood (Montgomery 2010).

### Implications for Theory

Although the lack of a statistically significant difference between work-themed play and work at complex activities is inconsistent with our hypothesis, we argue that our findings nonetheless support the embodied capital theory. Proponents of the adult mortality model have argued that body size, and not knowledge, restricts children from participating fully in subsistence activities. For example, Bird and Bliege Bird (2005) found that height and walking speed were better predictors of Martu children’s hunting success than age, and that, after age five, age alone has little effect on hunting success for goannas in rocky outcrops. The authors suggested that children make optimal foraging choices based on constraints restricted by body size, and not on learned skill. Though this may be true for smaller game, which are more abundant and thus have a higher encounter rate, several studies have found that success at hunting large game is independent of strength and size (Crittenden et al. 2013; Gurven et al. 2006; Ohtsuka 1989; Walker et al. 2002). For example, Crittenden et al. (2013) found that Hadza children successfully collect small game, birds, and fruit, but none of the children included in their sample ever successfully collected big game. Among the Gidra, both Ohtsuka (1989) and Kawabe (1983) have demonstrated that strength, size, and target shooting does not predict hunting success, whereas environmental knowledge does. Thus, it may be encounter rate, and not body size, which affects children’s foraging success. Play might be a viable way to practice when encounter rates are low, as they are in the Congo Basin tropical forests. By learning through play, children develop the knowledge they need to increase their encounter rate through tracking and stalking. These findings support the embodied capital theory and suggest work-themed play is an important way through which children develop skill and knowledge.

However, not all of our findings are consistent with the embodied capital theory; the fact that the children in this study were found to spend more time working on simpler tasks at all ages than playing at them—and far more time on these tasks then they spent on complex tasks—does not lend clear support to either model of the evolution of childhood. Based on the data presented here, Aka and Ngandu children were easily able to perform many essential, if “simple,” subsistence tasks (i.e., gathering, food preparation, commerce, miscellaneous chores) from as young as four years of age, suggesting they were not necessarily restricted by either body size or knowledge. In other words, little embodied capital investment was needed. At the same time, if play is not an adaptation for learning, we might expect children to play more than they work at all tasks, since play by definition is associated with positive affect (Smith 2010). In order to make sense of these inconsistencies, we must turn to the gender-typical and ethno-typical nature of play described above; we argue here that, for humans, play not only contributes to the development of embodied capital but is also part of an evolved culture-learning psychology which provides an intrinsic motivation to participate in the subsistence economy alongside other aspects of culture (Boyette 2016a; Rogoff et al. 2003). We offer two lines of evidence in support of this argument.

First, although both Aka and Ngandu children are commonly told to perform certain tasks, only the Ngandu children are coerced into fulfilling their responsibilities through shaming and threats of physical punishment; Aka children are rarely coerced (Boyette and Hewlett 2017). Thus, at least for the Aka, and we believe for the Ngandu as well, the movement from pretense play to work makes participating in work—or performing a dance or being initiated—when one is able just as rewarding as pretending to do so. Secondly, as Crittenden (2016) argues, making a distinction between work and play obscures their inherent duality when it comes to work-themed pretense. Consistent with this view is that of Tucker and Young (2005), who observed Mikea children throwing away edible tubers in “food fights” during foraging. They concluded from this observation that, at least for forager children, foraging is an extension of play.

We do not find that the work presented here or that of Bock and Johnson, for example, is inconsistent with this view. Rather, the evidence suggests that as children mature, “work-themed play” comes to resemble “work.” The age at which an observer might identify the trade-off from one to the other—by measuring caloric returns or through other means—is influenced by the natural and cultural ecology in which the children are reared, and therefore also upon the diversity of “simple” and “complex” resources that are available. For example, Blurton Jones and colleagues’ classic comparison of foraging returns of the Hadza versus the !Kung (Blurton Jones et al. 1989, 1994) demonstrated that the rocky woodland ecology in which Hadza children lived was far more amenable to children’s active foraging than was the flat, featureless Kalahari, which demanded many more years of experience to successfully navigate. This body of research, including the current study, suggests to us that the very variability of human subsistence and culture strongly supports the idea that play is a flexible learning adaptation, and that its concentration in childhood is no coincidence, but that childhood itself is a result of selection to prepare children for the task of survival and reproduction in a complex, variable social and subsistence ecology.

## Conclusion

Using behavioral observation data of Aka and Ngandu children from the Central African Republic, this paper has attempted to answer two main questions: Do children choose play and work activities that reflect the gender and culture norms within their communities? And, do children spend more time in play at activities that are too complex to learn “on the job”? Our findings show that ethnic and gender biases are apparent in the work and work-themed play behavior of forager children in their everyday settings. Furthermore, this paper is the first to document ethno-typical play in a multiethnic community of farmers and foragers. Finally, our results also further support Bock and Johnson’s (2004) hypothesis that play helps children learn complex skills.

This paper has several limitations. First, the definitions presented here for work and work-themed play did not determine the contributions children were actually making toward the household. When children are working, are they actually offsetting their costs, or are they merely practicing? Measuring children’s foraging returns alongside their participation in work and play can provide further insight into the embodied capital hypothesis. Second, because of the small sample sizes and poor data distribution, only nonparametric tests could be conducted for the activity categories. Complex interactions regarding how age, gender, and ethnicity influence each activity type could not be explored. Despite these limitations, this paper has highlighted the importance of considering work-themed play as one of the various ways in which children develop their embodied capital and also makes important, parallel contributions to developing culturally appropriate behaviors. Future studies will explore how gender and ethnic biases develop, and how time spent in work and work-themed play and children’s foraging returns interact as part of an ecology of learning.

## References

[CR1] Altmann, J. (1974). Observational study of behavior: Sampling methods. *Behaviour, 49*(3/4), 227–267. 10.1163/156853974x00534 4597405

[CR2] Bahuchet S, De Garine I, Harrison G (1988). Food supply uncertainty among the Aka pygmies (Lobaye, Central African Republic). Coping with uncertainty in food supply.

[CR3] Bateson P (2014). Playfulness and creativity. Animal Behavior and Cognition.

[CR4] Beach FA (1945). Current concepts of play in animals. The American Naturalist.

[CR5] Bekoff M, Byers JA, Immelmann K, Barlow G, Main M, Petrinovich L (1981). A critical reanalysis of the ontogeny and phylogeny of mammalian social play: An ethological hornet’s nest. Behavioural development in animals and man.

[CR6] Berry, J. W., Bahuchet, S., van De Koppel, J. M. H., Annis, R., Senechal, C., Cavalli-Sforza, L. L., and Witkin, H. (1986). *On the edge of the forest: Cultural adaptation and cognitive development in Central Africa.* Lisse: Swets and Zeitlinger.

[CR7] Bird DW, Bliege Bird R, Hewlett BS, Lamb ME (2005). Martu children’s hunting strategies in the Western Desert, Australia. Hunter-gatherer childhoods: Evolutionary, developmental and cultural perspectives.

[CR8] Bliege Bird R, Bird DW (2002). Constraints of knowing or constraints of growing? Fishing and collecting by the children of Mer. Human Nature.

[CR9] Blurton Jones N, Marlowe FW (2002). Selection for delayed maturity: Does it take 20 years to learn to hunt and gather?. Human Nature.

[CR10] Blurton Jones N, Hawkes K, O’Connell JF, Standen V, Foley R (1989). Modelling and measuring costs of children in two foraging societies. Comparative socioecology: The behavioural ecology of humans and other mammals.

[CR11] Blurton Jones NG, Hawkes K, Draper P (1994). Foraging returns of !Kung adults and children: Why didn’t !Kung children forage?. Journal of Anthropological Research.

[CR12] Bock J (2002). Learning, life history, and productivity: Children’s lives in the Okavango Delta, Botswana. Human Nature.

[CR13] Bock J, Pellegrini AD, Smith PK (2005). Farming, foraging, and children’s play in the Okavango Delta, Botswana. The nature of play: Great apes and humans.

[CR14] Bock J, Johnson SE (2004). Subsistence ecology and play among the Okavango Delta peoples of Botswana. Human Nature.

[CR15] Boesch C, Bombjaková D, Boyette AH, Meier A (2017). Technical intelligence and culture: Nut cracking in humans and chimpanzees. American Journal of Physical Anthropology.

[CR16] Bornstein M, Göncü A, Gaskins S (2006). On the significance of social relationships in the development of children’s earliest symbolic play: An ecological perspective. Play and development: Evolutionary, sociocultural, and functional perspectives.

[CR17] Boyette, A. H. (2013). *Social learning during middle childhood among Aka forest foragers and Ngandu farmers of the Central African Republic*. PhD dissertation, Washington State University, Pullman.

[CR18] Boyette AH (2016). Children’s play and culture learning in an egalitarian foraging society. Child Development.

[CR19] Boyette AH, Terashima H, Hewlett BS (2016). Children’s play and the integration of social and individual learning: A cultural niche construction perspective. Social learning and innovation in contemporary hunter-gatherers: Evolutionary and ethnographic perspectives.

[CR20] Boyette AH, Hewlett BS (2017). Autonomy, equality, and teaching among Aka foragers and Ngandu farmers of the Congo Basin. Human Nature.

[CR21] Buttelmann D, Zmyj N, Daum M, Carpenter M (2013). Selective imitation of in-group over out-group members in 14-month-old infants. Child Development.

[CR22] Byers JA, Walker C (1995). Refining the motor training hypothesis for the evolution of play. The American Naturalist.

[CR23] Charnov EL (1993). Life history invariants.

[CR24] Charnov EL, Berrigan D (1993). Why do female primates have such long lifespans and so few babies? Or, life in the slow lane. Evolutionary Anthropology.

[CR25] Chick G, Lancy DF, Bock J, Gaskins S (2009). Work, play and learning. The anthropology of learning in childhood.

[CR26] Chisholm JS, Ellison PT, Evans J, Lee PC, Lieberman LS, Pavlik Z (1993). Death, hope, and sex: Life-history theory and the development of reproductive strategies [and comments and reply]. Current Anthropology.

[CR27] Crittenden, A. N. (2016). Children’s foraging and play among the Hadza: The evolutionary significance of “work play.” In C. L. Meehan and A. N. Crittenden (Eds.), *Childhood: Origins, evolution and implications* (pp. 155–170). Santa Fe: SAR Press.

[CR28] Crittenden AN, Conklin-Brittain NL, Zes DA, Schoeninger MJ, Marlowe FW (2013). Juvenile foraging among the Hadza: Implications for human life history. Evolution and Human Behavior.

[CR29] Dira S, Hewlett BL, Takada A, Hewlett BS (2016). Learning to spear hunt among Ethiopian Chabu adolescent hunter-gatherers. Social learning and innovation in contemporary hunter-gatherers: Evolutionary and ethnographic perspectives.

[CR30] Draper P (1975). Cultural pressure on sex differences. American Ethnologist.

[CR31] Draper P, Cashdan E (1988). Technological change and child behavior among the !Kung. Ethnology.

[CR32] Ember C, Cunnar C (2015). Children’s play and work: The relevance of cross-cultural ethnographic research for archaeologists. Childhood in the Past.

[CR33] Endicott KM, Endicott KL (2008). The headman was a woman: The gender egalitarian Batek of Malaysia.

[CR34] Fagen R (1981). Animal play behavior.

[CR35] Flannery R (1953). The Gros Ventres of Montana, part 1: Social life.

[CR36] Flynn EG, Laland KN, Kendal RL, Kendal JR (2013). Developmental niche construction. Developmental Science.

[CR37] Fouts HN, Hallam RA (2013). Gender segregation in early-childhood social play among the Bofi foragers and farmers in Central Africa. American Journal of Play.

[CR38] Fouts HN, Lamb ME (2009). Cultural and developmental variation in toddlers’ interactions with other children in two small-scale societies in Central Africa. *International Journal of Developmental*. Sciences.

[CR39] Fouts HN, Bader LR, Neitzel CL (2016). Work-themed play among young children in foraging and farming communities in Central Africa. Behaviour.

[CR40] Gallois S, Duda R, Hewlett BS, Reyes-García V (2015). Children’s daily activities and knowledge acquisition: A case study among the Baka from southeastern Cameroon. Journal of Ethnobiology and Ethnomedicine.

[CR41] Garfield ZH, Garfield MJ, Hewlett BS, Terashima H, Hewlett BS (2016). A cross-cultural analysis of hunter-gatherer social learning. Social learning and innovation in contemporary hunter-gatherers: Evolutionary and ethnographic perspectives.

[CR42] Gaskins S (2000). Children’s daily activities in a Mayan village: A culturally grounded description. Cross-Cultural Research.

[CR43] Gosso Y, e Morais M d LS, Otta E (2007). Pretend play of Brazilian children: A window into different cultural worlds. Journal of Cross-Cultural Psychology.

[CR44] Gray P (2009). Play as a foundation for hunter-gatherer social existence. American Journal of Play.

[CR45] Gurven M, Kaplan HS, Gutierrez M (2006). How long does it take to become a proficient hunter? Implications for the evolution of extended development and long life span. Journal of Human Evolution.

[CR46] Hawkes K (2003). Grandmothers and the evolution of human longevity. American Journal of Human Biology.

[CR47] Hawkes K, O’Connell JF, Blurton Jones NG (1995). Hadza children’s foraging: Juvenile dependency, social arrangements, and mobility among hunter-gatherers. Current Anthropology.

[CR48] Hawkes K, O’Connell JF, Jones NGB, Alvarez H, Charnov EL (1998). Grandmothering, menopause, and the evolution of human life histories. Proceedings of the National Academy of Sciences.

[CR49] Henrich J, McElreath R (2003). The evolution of cultural evolution. Evolutionary Anthropology.

[CR50] Hewlett BS (1991). Intimate fathers: The nature and context of Aka pygmy paternal infant care.

[CR51] Hewlett BS, Cavalli-Sforza LL (1986). Cultural transmission among Aka pygmies. American Anthropologist.

[CR52] Hewlett BL, Hewlett BS, Hewlett BL (2012). Hunter-gatherer adolescence. Adolescent identity: Evolutionary, cultural and developmental perspectives.

[CR53] Hewlett B, Roulette J, Narvaez D, Valentino K, Fuentes A, McKenna J, Gray P (2014). Cosleeping beyond infancy. Ancestral landscapes in human evolution: Culture, childrearing and social wellbeing.

[CR54] Hewlett BS, Fouts HN, Boyette AH, Hewlett BL (2011). Social learning among Congo Basin hunter-gatherers. Philosophical Transactions of the Royal Society, B: Biological Sciences.

[CR55] Imamura K, Terashima H, Hewlett BS (2016). Hunting play among the San children: Imitation, learning, and play. Social learning and innovation in contemporary hunter-gatherers: Evolutionary and ethnographic perspectives.

[CR56] Kaplan HS (1996). A theory of fertility and parental investment in traditional and modern human societies. Yearbook of Physical Anthropology.

[CR57] Kaplan HS, Robson AJ (2002). The emergence of humans: The coevolution of intelligence and longevity with intergenerational transfers. Proceedings of the National Academy of Sciences.

[CR58] Kaplan HS, Hill K, Lancaster J, Hurtado AM (2000). A theory of human life history evolution: Diet, intelligence, and longevity. Evolutionary Anthropology.

[CR59] Kaplan HS, Lancaster J, Robson A (2003). Embodied capital and the evolutionary economics of the human life span. Population and Development Review.

[CR60] Kawabe T (1983). Development of hunting and fishing skill among boys of the Gidra in lowland Papua New Guinea. Journal of Human Ecology.

[CR61] Konner MJ, Hewlett BS, Lamb ME (2005). Hunter-gatherer infancy and childhood: The !Kung and others. Hunter-gatherer childhoods: Evolutionary, developmental and cultural perspectives.

[CR62] Konner MJ (2010). The evolution of childhood: Relationships, emotion, mind.

[CR63] Kramer KL (2002). Variation in juvenile dependence: Helping behavior among Maya children. Human Nature.

[CR64] Lancy DF (1996). Playing on the mother-ground: Cultural routines for children’s development.

[CR65] Lancy DF (2008). The anthropology of childhood.

[CR66] Lancy DF, Bourdillon MFC, Spittier G (2012). The chore curriculum. African children at work: Working and learning in growing up for life.

[CR67] Lancy DF (2016). New studies of children’s work, acquisition of critical skills, and contribution to the domestic economy. Ethos.

[CR68] Lancy DF, Grove MA (2011). Getting noticed: Middle childhood in cross-cultural perspective. Human Nature.

[CR69] Leigh SR (2001). Evolution of human growth. Evolutionary Anthropology.

[CR70] Lewis, J. (2002). *Forest hunter-gatherers and their world: A study of Mbendjele Yaka pygmies of Congo-Brazzaville and their secular and religious activities and representations*. PhD dissertation, London School of Economics and Political Science.

[CR71] Lew-Levy S, Reckin R, Lavi N, Cristóbal-Azkarate J, Ellis-Davies K (2017). How do hunter-gatherer children learn subsistence skills? A meta-ethnographic review. Human Nature.

[CR72] Lew-Levy S, Lavi N, Reckin R, Cristóbal-Azkarate J, Ellis-Davies K (2018). How do hunter-gatherer children learn social and gender norms? A meta-ethnographic review. Cross-Cultural Research.

[CR73] Lillard AS (1993). Pretend play skills and the child’s theory of mind. Child Development.

[CR74] Little CAJL, Lancy DF (2016). How do children become workers? Making sense of conflicting accounts of cultural transmission in anthropology and psychology. Ethos.

[CR75] Long SJ, Freese J (2006). Regression models for categorical dependent variables using Stata.

[CR76] MacDonald K (2007). Cross-cultural comparison of learning in human hunting: Implications for life history evolution. Human Nature.

[CR77] Marlowe FW (2007). Hunting and gathering: The human sexual division of foraging labor. Cross-Cultural Research.

[CR78] Montgomery H, Lancy DF, Bock J, Gaskins S (2010). Learning gender roles. The anthropology of learning in childhood.

[CR79] Morelli GA, Morbeck ME, Galloway A, Zihlman A (1997). Growing up female in a farmer community and a forager community. The evolving female: A life history perspective.

[CR80] Munroe RH, Munroe RL, Shimmin HS (1984). Children’s work in four cultures: Determinants and consequences. American Anthropologist.

[CR81] Nag M, White BNF, Peet RC, Bardhan A, Terence H (1978). An anthropological approach to the study of economic value of children in Java and Nepal. Current Anthropology.

[CR82] Neuwelt-Truntzer, S. (1981). *Ecological influences on the physical, behavioral, and cognitive development of pygmy children*. PhD dissertation, University of Chicago.

[CR83] O’Connell JF, Hawkes K, Blurton Jones NG, Ungar P, Teaford M (2002). Meat-eating, grandmothering and the evolution of early human diets. Human diet: Its origin and evolution.

[CR84] Ohtsuka R (1989). Hunting activity and aging among the Gidra Papuans: A biobehavioral analysis. American Journal of Physical Anthropology.

[CR85] Over H, Carpenter M (2013). The social side of imitation. Child Development Perspectives.

[CR86] Pellegrini AD, Bjorklund DF (2004). The ontogeny and phylogeny of children’s object and fantasy play. Human Nature.

[CR87] Rogoff B, Paradise R, Arauz RM, Correa-Chávez M, Angelillo C (2003). Firsthand learning through intent participation. Annual Review of Psychology.

[CR88] Roscoe P (2006). Fish, game, and the foundations of complexity in forager society: The evidence from new Guinea. Cross-Cultural Research.

[CR89] Rupp S, Hewlett BS (2014). Multiangular identities among Congo River Basin forest people. Hunter-gatherers of the Congo Basin: Cultures, histories, and biology of African pygmies.

[CR90] Shostak M, Lee RB, DeVore I (1976). A !Kung woman’s memories of childhood. Kalahari hunter-gatherers: Studies of the !Kung San and their neighbors.

[CR91] Slaughter D, Dombrowski J, Bloch M, Pellegrini AD (1989). Cultural continuities and discontinuities: Impact on social and pretend play. The ecological context of children’s play.

[CR92] Smith PK (1982). Does play matter? Functional and evolutionary aspects of animal and human play. Behavioral and Brain Sciences.

[CR93] Smith PK (2010). Children and play.

[CR94] Thomas JMC, Bahuchet S (1991). Encyclopédie des pygmée Aka: Techniques, langage et société de chasseurs-cueilleurs de la forêt centrafricaine (Sud-Centrafrique et Nord-Congo).

[CR95] Tucker B, Young AG, Hewlett BS, Lamb ME (2005). Growing up Mikea: Children’s time allocation and tuber foraging in southwestern Madagascar. Hunter-gatherer childhoods: Evolutionary, developmental and cultural perspectives.

[CR96] Turnbull CM (1962). The forest people.

[CR97] Turnbull, C. M. (1978). The politics of non-aggression. In A. Montagu (Ed.), *Learning non-aggression: The experience of non-literate**societies* (pp. 161–211). Oxford: Oxford University Press.

[CR98] Vanstone JW (1965). The changing culture of the Snowdrift Chipewyan.

[CR99] Walker R, Hill K, Kaplan HS, McMillan G (2002). Age-dependency in hunting ability among the Ache of eastern Paraguay. Journal of Human Evolution.

[CR100] Wallace E, Hoebel EA (1952). The Comanches: Lords of the South Plains.

[CR101] Whiting BB, Edwards CP (1973). A cross-cultural analysis of sex differences in the behavior of children aged 3 through 11. Journal of Social Psychology.

[CR102] Whiting BB, Whiting JWB (1975). Children of six cultures: A psycho-cultural analysis.

